# Sex hormones affect neurotransmitters and shape the adult female brain during hormonal transition periods

**DOI:** 10.3389/fnins.2015.00037

**Published:** 2015-02-20

**Authors:** Claudia Barth, Arno Villringer, Julia Sacher

**Affiliations:** ^1^Department of Neurology, Max Planck Institute for Human Cognitive and Brain SciencesLeipzig, Germany; ^2^Clinic of Cognitive Neurology, University of LeipzigLeipzig, Germany; ^3^Leipzig Research Center for Civilization Diseases, University of LeipzigLeipzig, Germany; ^4^Integrated Research and Treatment Center Adiposity Diseases, University of LeipzigLeipzig, Germany; ^5^Berlin School of Mind and Brain, Mind and Brain InstituteBerlin, Germany

**Keywords:** estrogens, progesterone, neurotransmitters, plasticity, hormonal transition periods

## Abstract

Sex hormones have been implicated in neurite outgrowth, synaptogenesis, dendritic branching, myelination and other important mechanisms of neural plasticity. Here we review the evidence from animal experiments and human studies reporting interactions between sex hormones and the dominant neurotransmitters, such as serotonin, dopamine, GABA and glutamate. We provide an overview of accumulating data during physiological and pathological conditions and discuss currently conceptualized theories on how sex hormones potentially trigger neuroplasticity changes through these four neurochemical systems. Many brain regions have been demonstrated to express high densities for estrogen- and progesterone receptors, such as the amygdala, the hypothalamus, and the hippocampus. As the hippocampus is of particular relevance in the context of mediating structural plasticity in the adult brain, we put particular emphasis on what evidence could be gathered thus far that links differences in behavior, neurochemical patterns and hippocampal structure to a changing hormonal environment. Finally, we discuss how physiologically occurring hormonal transition periods in humans can be used to model how changes in sex hormones influence functional connectivity, neurotransmission and brain structure *in vivo*.

## Introduction

Over the last decades, several lines of research have extended the pivotal actions of ovarian hormones such as estrogen and progesterone outside of the reproductive tract. The brain represents an important target for estrogen and progesterone effects. Both hormones provide specific neuroendocrine conditions through which brain structure and function are modulated across a woman's life span. The trophic effects of ovarian hormones emerge early in brain development and remain throughout adolescence (Juraska et al., 2013) and adulthood (Wise et al., 2008). Many of these actions occur in brain regions involved in learning (Hu et al., 2007) and memory (Liu et al., 2008), emotion (Amin et al., 2006), motivation (Sakaki and Mather, 2012), motor control (Horstink et al., 2003), and cognition (Berman et al., 1997). Furthermore, several lines of evidence support a main impact of sex hormones on brain development and plasticity (Marino et al., 2006). Specific structural effects of estrogen and progesterone include neurite outgrowth and synaptogenesis (Haraguchi et al., 2012), dendritic branching (Cooke and Woolley, 2005) and myelination (Garcia-Segura and Melcangi, 2006).

### General mechanisms

Both estrogen and progesterone act via classical genomic receptors as well as non-classical membrane-associated receptors (see Table S1 for overview on main genomic and non-genomic signaling properties). The classical estrogen receptors (ERα/β) (Gundlah et al., 2001; Mitra et al., 2003) and progesterone receptors (PR_A/B_) (Brinton et al., 2008) are highly expressed in brain areas involved in emotion and cognition, such as amygdala and hippocampus. Ovarian hormones can act on multiple receptor types, such as voltage-gated ion channels, including GABA_A_ (Gulinello et al., 2001), NMDA (Foy et al., 1999), serotonin (Sumner and Fink, 1998) and dopamine (Becker, 1990) receptors. While these genomic actions of sex hormones require a comparably long time—from minutes to hours—and are limited by the rate of protein biosynthesis, non-genomic modulation of the membrane receptors is mostly faster and requires only milliseconds to seconds (McEwen, 1991; Cornil et al., 2006). Both estrogen and progesterone exert acute effects on synaptic physiology through the activation of multiple intracellular signaling pathways (Minami et al., 1990; Krebs et al., 2000; Wu et al., 2005), including the MAPK/ERK and the Akt pathway which are both part to a non-genomic signaling cascade linked to the promotion of cell survival (Singh, 2001). A distinct progesterone-binding protein different from the classical PR was identified as a membrane protein, known as 7TMPR, which mediates non-genomic actions via second-messenger cascades (Zhu et al., 2003a,b). However, genomic and non-genomic actions of hormones may also be coupled, so the distinctions are not as clear-cut as was first thought (Vasudevan and Pfaff, 2008).

It is noteworthy that most cellular effects of ovarian hormones have important roles in cell survival, apoptosis, function, and brain development and may act as critical neuroregulatory, neurotropic, and neuroprotective factors in brain physiology and pathological conditions of the brain. Effects on structure and function of the brain have been documented.

### Effects on structure

For instance, fairly recent studies have shown that progesterone, progestin or progestin metabolites could have the capacity to induce or inhibit neuroplastic changes by preventing microglia from releasing harmful free radicals (Muller and Kerschbaum, 2006) or by stimulating myelin production (Baulieu and Schumacher, 2000). Furthermore, ovarian hormones also exhibit profound effect on neurotrophins such as brain-derived neurotrophic factor (BDNF). BDNF has been shown to play a key role in neuronal survival, in promoting neuronal regeneration following injury and regulating neurotransmitter system (Scharfman and MacLusky, 2006; Sohrabji and Lewis, 2006). Estrogen treatment seems to increase BDNF expression in several brain regions including hippocampus, amygdala and cortex (Zhou et al., 2005), and has been shown to decrease the risk for neurodegenerative diseases such as Parkinson's disease and Alzheimer's disease (Sohrabji and Lewis, 2006).

However, much translational work remains to be done, and it is not clear which of these mechanisms are most relevant to clinical use. As an example, evidence for the beneficial effects of progesterone on cognitive outcome following traumatic brain injury has recently been reviewed as promising (Ma et al., 2012)—emphasizing the need to further promote this research direction.

Although estrogen and progesterone target multiple regions in the brain (Brinton et al., 2008), one brain region that has been the focus of many studies investigating potential neurotropic effects of these hormones is the hippocampus, a brain region associated with various memory functions (Bliss and Collingridge, 1993; Adams et al., 2004). Both, acute estrogen and progesterone treatment have been shown to increase synapse density and spine formation in hippocampal structures in rodents, respectively (Woolley and McEwen, 1993). However, the generative effects of progesterone seem to disappear after chronic treatment. Furthermore, progesterone has also been shown to down-regulate estrogen-induced synapses when added to estrogen-administration chronically (Woolley and McEwen, 1993). Thus, the duration and combination of ovarian hormone supplementation seems to be essential for its neuroplastic effects on brain structures, such as the hippocampus. Therefore, the overall modulatory effect of ovarian hormones is more complex than simple mechanistic processes of up- and down-regulation of expression patterns in isolated brain regions.

In humans, evidence for hormone-dependent modulatory effects on brain structure stems from hormonal replacement therapy (HRT) studies. The importance of ovarian hormones has lead to its use as HRT, primarily to treat menopausal/postmenopausal symptoms such as hot flashes and night sweat. However, structural and functional changes associated with HRT regimes have sparked a heightened interest in ovarian hormone effects in the human body. For instance, brain structure changes due to HRT seem to be most prominent in the hippocampus. Women using HRT showed an increased hippocampal volume compared to men and women who never used HRT (Lord et al., 2008).

Not only are exogenous sex steroid hormones likely to influence the structure and function of the hippocampus, but variable sex steroid levels across the female lifespan have also been associated with alterations in hippocampal structure (Adams et al., 2004; Galea et al., 2008).

### Effects on function

Beyond structural changes mediated by HRT, ovarian hormone supplementation is also known to have prominent effects on mood and cognitive functioning in domains such as working memory and executive control. In general, positive (Hogervorst et al., 2000; LeBlanc et al., 2001; Rice and Morse, 2003; Weber et al., 2014) and negative (Rapp et al., 2003; Shumaker et al., 2003) effects on cognition have been reported for HRT. In virtue of these contradictory findings, HRT is currently much under debate. However, it seems that timing (MacLennan et al., 2006) and dose (Rice, 2002) are critical aspects of how the impact of HRT unfolds.

A woman's lifespan is characterized by major hormonal transition periods beginning with rising estrogen level during puberty (Angold et al., 1998), high estrogen levels during pregnancy and rapid falls postpartum (Galea et al., 2001), declining levels during perimenopause (Cohen et al., 2006a) and low levels postmenopausal. Intriguingly, these major shifts in sex hormone levels seem to be paralleled by the incidence rates of mood disorders such as unipolar depression (**Figure 2**). According to the monoamine hypotheses of depression (Hindmarch, 2002), depressed mood seems to be accompanied by alternations in neurotransmitter functioning and transmission. Ovarian hormones are known to exhibit modulatory effects on synaptic transmission. These modulatory effects can be achieved by altering the responsiveness of postsynaptic receptors (Yankova et al., 2001; Maejima et al., 2013) or the presynaptic release of neurotransmitters (Yokomaku et al., 2003). The alternation of both mechanisms largely affect the neurochemical systems involved in healthy emotional and cognitive control, such as dopaminergic, serotonergic, glutamatergic and γ-aminobutyric acid (GABA)-ergic systems.

Along with the major hormonal transition periods, subtle changes in endogenous sex hormones, as occur during the monthly cycle, have also been associated with changes in mood (Backstrom et al., 2014). A subgroup of women, however, suffers from clinical level of premenstrual mood changes called premenstrual dysphoric disorder (PMDD), a condition that has recently been included in the DSM-V (Epperson et al., 2012a). PMDD core symptoms include anxiety, irritability and depressed mood (Epperson et al., 2012a). As the absolute levels of ovarian hormones do not seem to differ significantly in PMDD women and healthy controls (Backstrom et al., 2003), one hypothesis proposes that it is a heightened vulnerability of the central nervous system to normal ovarian function and physiological changes (Schmidt et al., 1998; Huo et al., 2007) rather than hormone imbalance, that predisposes women to PMDD. In the pathology of PMDD, the normal functioning of predominantly two main neurotransmitter system, the serotonergic and dopaminergic system seems to be impaired.

The present review aims to summarize recent findings on the interaction between ovarian hormones and various neurotransmitter systems in the brain. The second part of the review focuses on the discussion of these findings in the context of neuropsychiatric diseases that display a substantial sexual dimorphism, such as affective disorders and Alzheimer's disease.

## Sex hormones and their interaction with neurotransmitter systems—potential mechanisms for sex hormones to effect brain structure and function

The classification of the main neurotransmitter systems are summarized in Table S1.

### Sex hormones and glutamate interaction

**Figure 1:**
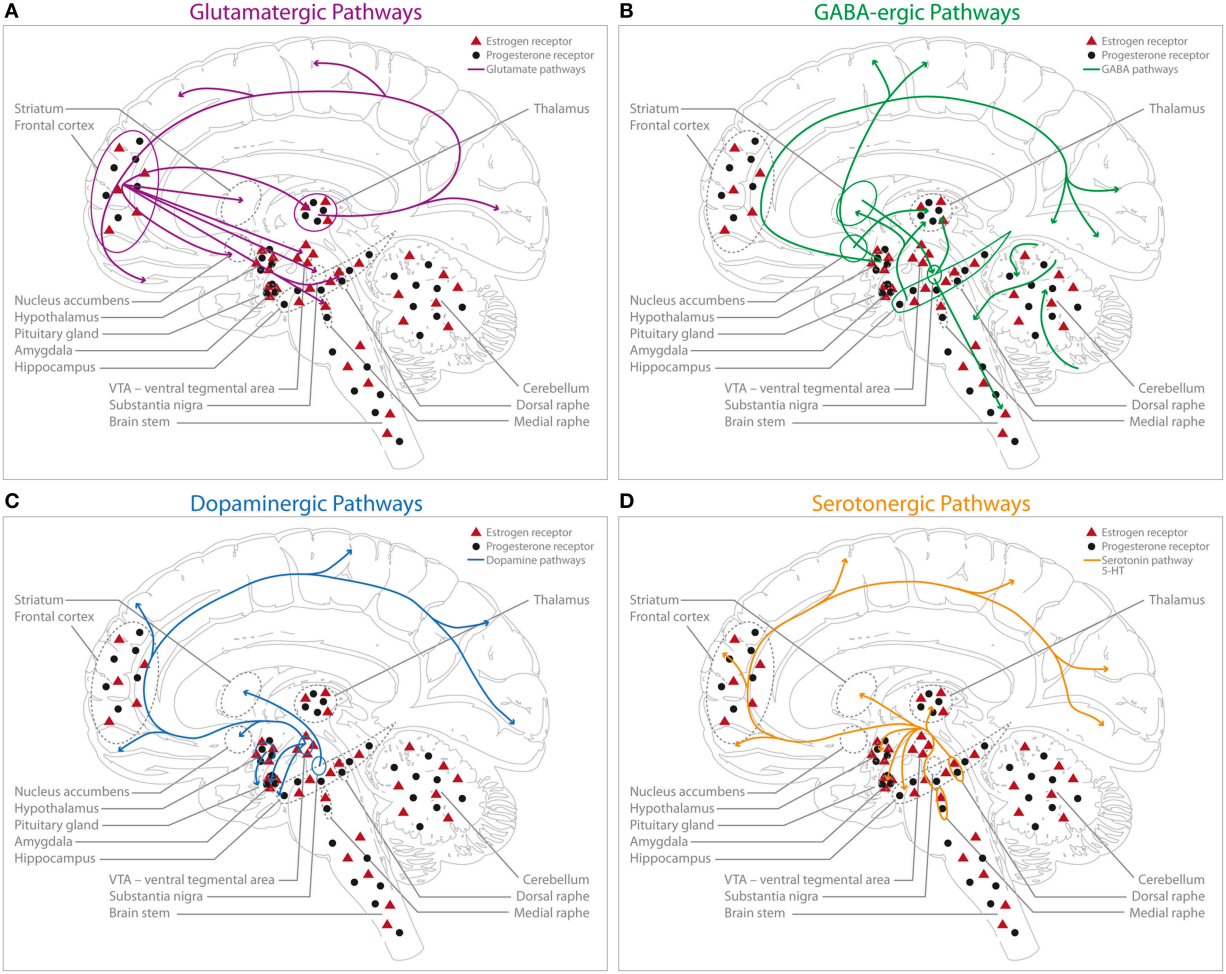
**Schematic representation of the main human central glutamatergic system (A, purple), GABAergic system (B, green), dopaminergic system (C, blue) and serotonergic system (D, orange) including a schematic display of estrogen (ERα and ERβ combined) and progesterone receptor distribution in the human brain**. No distinction is made between ERα and ERβ sub-specification for this schematic display, however the localization for those subtypes can differ [e.g., so far no evidence could be gathered supporting ERα expression in the dorsal raphe nucleus (DRN) (Sugiyama et al., 2010)], however there have been reports on ERβ expression in primate DRN in the midbrain (Gundlah et al., 2001; Sugiyama et al., 2010). Estrogen (red triangles) receptors are predominantly present in cerebellum, VTA, hippocampus, amygdala, and frontal cortex; as well as in the raphe nuclei of the midbrain (Gundlah et al., 2001; Osterlund and Hurd, 2001; Mitra et al., 2003; Perlman et al., 2005; Sugiyama et al., 2010). Progesterone (filled circles) receptor expression could be shown in the amygdala, midbrain, brain stem, hippocampus, cerebellum and frontal cortex with no apparent restrictions to specific cell types (Bethea, 1993; Gundlah et al., 2001). **(A)** The cortical glutamatergic projections can be separated in five main pathways (Schwartz et al., 2012): (1) from prefrontal to brainstem areas (dorsal/medial raphe, VTA, substantia nigra); (2) from prefrontal cortex to striatum and nucleus accumbens; (3) the thalamocortical pathway, from thalamus to cortical pyramidal neurons; (4) inverse projections from cortex to thalamus; and (5) intra-cortical glutamate projections. **(B)** GABAergic projections are widely distributed throughout the brain. Main projections can be found originating in the striatum to the substantia nigra and the brain stem. Further projections innervate the thalamus from the substantia nigra (Fino and Venance, 2010). Moreover, GABAergic projections originate from (1) hypothalamus to occipital cortex and parietal cortex; (2) from hippocampus to thalamus and striatum, and (3) from nucleus accumbens to thalamus. The cerebellum is also highly innervated by GABAergic projections. **(C)** The cortical dopaminergic pathways build four distinct pathways (Felten and Shetty, 2010): (1) the mesolimbic pathway; with projections from VTA to limbic structures, such as the nucleus accumbens, hippocampus, amygdala and prefrontal cortex, (2) the mesocortical pathway; with projections from the VTA to cerebral cortex, (3) the nigrostriatal pathway; with connections between substantia nigra and striatum, (4) the tuberoinfundibular pathway; with projections starting from the hypothalamus to the pituitary gland. **(D)** The majority of serotonergic projections originates from the dorsal and median raphe nuclei, innervating the amygdala, hypothalamus, thalamus, striatum, cerebral cortex and hippocampus (Felten and Shetty, 2010): (1) The medial raphe predominantly projects to the frontal cortex and the hippocampus (Hornung, 2003) and (2) the areas of the dorsal raphe mainly innervate the thalamus, striatum and cerebral cortex (Geyer et al., 1976).

Glutamate acts as the main excitatory neurotransmitter in the CNS and is a proximal regulator of cognitive domains such as learning and memory (Gazzaley et al., 1996; Foy et al., 1999; Riedel et al., 2003). The integration of glutamatergic transmission is fundamental for normal cognitive functioning and mental health (Schwartz et al., 2012; Abdallah et al., 2014). The cortical glutamate projections are organized in descending and ascending pathways that project throughout much of the telencephalon (Figure 1A). The impact of ovarian hormones on the glutamatergic system has been studied extensively, especially in cell cultures (Yokomaku et al., 2003) and animal models (Bethea and Reddy, 2012; Wei et al., 2014). Both stimulatory (Yokomaku et al., 2003) and inhibitory (Smith et al., 1987a) effects of ovarian hormones have been reported. In rodents, several mechanisms through which ovarian hormones may influence glutamatergic neurotransmission have been proposed: progesterone has been shown to suppress the excitatory glutamate response in a dose-dependent fashion (Smith et al., 1987a), while estrogen exhibits facilitating effects on glutamate transmission (Yokomaku et al., 2003). A physiological dose of progesterone in ovariectomized rats has been reported to reduce glutamate-response by 87% via attenuation of non-NMDA receptors (AMPA, Kainate) (Smith et al., 1987b). The magnitude of the attenuation seems directly proportional to the progesterone dose. Whereas progesterone mainly impacts non-NMDA receptors (Smith et al., 1987a), the mechanisms underlying estrogen effects on cognition are related to NMDA glutamate receptors. Estrogen has been shown to promote an increase in NMDA receptor subunit expression (Gazzaley et al., 1996; Adams et al., 2004), binding sites (Woolley et al., 1997) and neuronal sensitivity to synaptic input mediated by NMDA glutamate receptors (Rudick and Woolley, 2001; Smith and Woolley, 2004). The blockade of NMDA receptors with antagonists attenuates the effects of estrogen on neuronal correlates of memory, such as long-term potentiation (LTP) (Brinton et al., 1997; Foy et al., 1999). Moreover, estrogen facilitates the spine-maturation process (Hao et al., 2006). A plethora of animal studies have shown that estrogen with and without progesterone increases dendritic spines through the up-regulation of AMPA (Liu et al., 2008; Kramar et al., 2009) and NMDA receptors (Woolley et al., 1997) in the hippocampus and prefrontal cortex (PFC) (Hao et al., 2006). In addition, ovariectomy reduced synaptic markers in these regions (Gould et al., 1990; Hao et al., 2006).

Beside ovarian influence on spine density, precursors for progesterone (such as pregnenolone) seem to have complex effects on glutamate release itself that depends on the developmental period, brain region and functional state (Zheng, 2009): in the hippocampus (Meyer et al., 2002) and in the prefrontal cortex (Dong et al., 2005), which are brain regions of high relevance to memory and executive control, progesterone precursors have been shown to impact spontaneous glutamate release that may contribute to the maturation and/or maintenance of synapses. The latter might point toward the well-established neuroprotective effects (Mattson et al., 1997; Wang et al., 2001) of ovarian hormones from insults such as oxidative stress and glutamate excitotoxicity.

In addition to these cellular effects, recent studies report that the interaction between estrogen and glutamate can affect cognitive domains such as working memory and executive function under harmful conditions. Brain regions hypothesized to underlie these cognitive domains—such as the PFC and hippocampus—seem largely dependent on normal estrogen signaling to counter insults such as stress: In a repeated stress paradigm, Wei et al. (2014) found a beneficial effect of endogenous estrogen on glutamate receptors in the PFC in female rats compared to male rats. The authors propose that detrimental effects of repeated stress are present in females when estrogen signaling is blocked, whereas detrimental effects are blocked in males when estrogen signaling is activated. In particular, blocking estrogen synthesis enzyme aromatase with formestane in PFC revealed stress-induced glutamatergic deficits and memory impairment in female rats (Wei et al., 2014). Thus, these results suggest that the female rodent PFC has an endogenous capacity to generate estrogen that provides protection against subchronic repeated stress. How gender differences in response to stressors are modulated by hormonal status is extensively reviewed by Cohen and Yehuda (2011). In addition to endogenous estrogen levels, also exogenous administered estrogen seems to increase the resilience to stress and preserve hippocampal functioning in rats (Bredemann and McMahon, 2014).

Although animal studies assessing electrophysiological, biochemical and behavioral markers for sex hormonal impacts on the glutamatergic system provide useful insights on underlying mechanisms, extrapolation to humans is difficult. Some of the beneficial effects of estrogen on cognitive function have also been shown in humans: premenopausal women who were treated with a gonadotropin releasing hormone analog which chemically suppressed ovarian function experienced significant deterioration of mood and worsening of performance in working memory tasks (Grigorova et al., 2006). In this study, the estrogen levels dropped to postmenopausal values implicating that low endogenous levels of estrogen might impair normal cognitive functioning.

Although this study report positive effects of estrogen on working memory and executive function, the results are inconclusive (Grigorova and Sherwin, 2006), and the advisability of hormonal replacement is still much under debate. However, a large number of studies with behavioral testing during hormonal transitions, such as the menstrual cycle or postmenopause, point toward an estrogen-dependent improvement in memory (Epperson et al., 2012b; Hampson and Morley, 2013), but the neurochemical pathways underlying these changes remain to be identified. A useful approach to assess glutamate release *in vivo* in humans might be a pharmacological stimulation of the glutamatergic system with positron emission tomography (PET) using a glutamate-receptor radioligand. In conjuction with MR-Imaging it could link glutamate release to brain activation during, i.e., working memory tasks. Such methods should lead to a better understanding of the interaction between sex hormones and glutamatergic neurotransmission.

### Sex steroid hormone and gamma-aminobutyric-acid interaction

Gamma-aminobutyric acid (GABA) is the most abundant and widely distributed inhibitory neurotransmitter in the CNS (Sieghart and Sperk, 2002; Marshall, 2008). GABAergic neurotransmission through interneurons is known to modulate local neuronal circuits via, for example, activation of dopaminergic (Dewey et al., 1992) and serotonergic neurons (Andrade et al., 1986). GABAergic interneurons can be differentiated into two types, each acting via its receptor-subtype (Table S2). GABA receptors are highly distributed in cortical, hippocampal, thalamic, basal ganglia and cerebellar structures (Figure 1B).

GABA_A_ receptors mediate major inhibitory GABAergic actions in the CNS and are putative sites for ovarian hormone effects (Backstrom et al., 2011, 2014). Whereas estrogen seems to suppress GABA inhibitory input (Murphy et al., 1998a), progesterone and its neuroactive metabolites (allopregnanolone, pregnanolone) seem to facilitate GABAergic transmission through their action at GABA_A_ receptors (van Wingen et al., 2008; Deligiannidis et al., 2013). Particularly, allopregnanolone acts like a positive modulator and potentiates the inhibitory action of GABA by increasing channel openings of the GABA-gated chloride channels (Rupprecht, 1997) and augmenting other inhibitory neuronal responses to GABA (Smith, 1991). This facilitation of GABA-mediated Cl^−^ current can result in inhibitory effects on neuronal function.

The interaction of progesterone and its neurosteroids with neuronal GABA_A_ receptors is significantly influenced by the subunit composition of the receptor, local metabolism and phosphorylation (Belelli et al., 2006; Backstrom et al., 2014). Especially the subunit composition seems to play a crucial role in inhibitory neurotransmission and its effects on a larger scale such as mood and cognition (Backstrom et al., 2014). Animal studies indicate a relationship between changes in α4 and δ subunits of GABA_A_ receptors and anxiogenic effects of allopregnanolone (Gulinello et al., 2001). Alternations in both GABA receptor subunit expression and anxiety behavior reflect a complex temporal pattern following sustained exposure to progesterone metabolites: An increase in hippocampal expression of the α4 subunit is seen to correlate with increased anxiety after 48 h exposure to allopregnanolone (Hsu et al., 2003). Paradoxically, in high concentrations, progesterone and its neurosteroids are also known to be anxiolytic, sedative, and antiepileptic, both in animals and humans (Backstrom et al., 2014). Allopregnanolone naturally fluctuates across the female menstrual cycle, with its highest concentration in the luteal phase when progesterone is high and estrogen is low (Backstrom et al., 2014). In women with PMDD, progesterone withdrawal associated with allopregnanolone increase in the luteal phase has been linked to changes in mood (Epperson et al., 2012a). As absolute levels of ovarian hormones do not seem to differ in PMDD women compared to healthy controls (Backstrom et al., 2003), it is proposed that a heightened vulnerability of the central nervous system to normal ovarian function predisposes women to PMDD.

Beyond its influence on mood, progesterone and its metabolites also seem to impact the memory and learning domains. Animal studies could show that allopregnanolone can inhibit neural activity in the CA1 and the dental gyrus area of the hippocampus (Landgren et al., 1998). The magnitude of the allopregnanolone inhibition is dependent on the phase of the rodent estrus cycle, with its maximum in the luteal phase. In humans, acute progesterone or allopregnanolone administration has been shown to impair face recognition and episodic memory in healthy women, while endogenous allopregnanolone level does not seem to impact their memory and learning. However, women suffering from PMDD showed impaired working memory in N2 and N3 back tasks during their symptomatic phase (Yen et al., 2012). Thus, healthy women seems to show memory impairments when progesterone and its metabolites are administered exogenously, while women with PMDD are more vulnerable to endogenous fluctuations of progesterone and allopregnanolone across the menstrual cycle.

Based on all these findings, Backstrom and colleagues recently hypothesized that an increase of the α4, β, δ subunit composition, together with a heightened vulnerability toward elevated allopregnanolone levels, could be key factors in the progesterone withdrawal model of PMDD (Backstrom et al., 2014). As PMDD heightens the risk for other kinds of depression, such as postpartum depression, a detailed understanding of the underlying mechanisms of PMDD might foster therapeutic approaches and thereby subsequently promote female mental health.

### Sex steroid hormone and dopamine interaction

Dopamine (DA) is a key neurotransmitter that is implicated in motor control (Sealfon and Olanow, 2000; Dluzen and Horstink, 2003), learning (Daniel et al., 2006), motivation (Becker, 2009), reward (Hikosaka et al., 2014), decision-making and working memory (Jacobs and D'Esposito, 2011; Uban et al., 2012). Brain areas that show rich dopaminergic innervation include the striatum, substantia nigra and hypothalamus (Sealfon and Olanow, 2000); typically four main pathways are described for the dopaminergic system (Figure 1C).

Sex hormones can impact dopaminergic neurotransmission via a multitude of mechanisms (synthesis, release, turnover and degradation, pre-and postsynaptic receptors, transporters, for details see Table S2). Stimulating (Becker, 1990, 2000; Thompson and Moss, 1994; Becker and Hu, 2008), as well as inhibiting (Disshon et al., 1998; Watson et al., 2006; Morel et al., 2009) effects of estrogen on dopaminergic neurotransmission have been documented. These conflicting findings are not surprising when considering the many aspects that can influence the impact of estrogen on the DA-system such as dose and time of testing, mode of administration, duration of exposure, and time after exposure (Di Paolo, 1994). Most experts agree that estrogen has an overall facilitating effect on dopaminergic neurotransmission (Sanchez et al., 2010; Jacobs and D'Esposito, 2011; Uban et al., 2012; Rey et al., 2014). For progesterone-modulating effects, it seems that, in addition to the previously mentioned aspects, priming with estrogen can also influence the impact of progesterone on dopaminergic transmission: *in vitro* (Dluzen and Ramirez, 1984; Cabrera et al., 1993) and *in vivo* (Becker, 1990) experiments support a stimulating effect on DA-release when rats where pre-exposed to estrogen whereas no such effects could be observed in non-estrogen treated rats.

Still much work remains to be done to improve our understanding of how these findings translate to the human brain, and ultimately link to human behavior and potentially pathology. Nevertheless, there are several examples for recent evidence converging from animal and human work that emphasize the relevance of this line of research to female mental health. Recent studies report on the interaction between estrogen and dopamine on cognitive domains, such as decision-making (Uban et al., 2012), fear extinction (Rey et al., 2014) and memory bias (Quinlan et al., 2013). The authors conclude that estradiol biases decision-making toward smaller, more accessible rewards (Uban et al., 2012), that a low-estrogen state during fear extinction is detrimental for an optimal freezing suppression after extinction, which is mediated by D1-receptor signaling (Rey et al., 2014) and that memory bias is mediated by the interaction of estradiol and dopamine in the dorsal striatum (Quinlan et al., 2013). In particular, the latter two studies argue that the effect of estrogen depends on individual variation in baseline DA function and endorse the concept of estrogen-dopamine interactions to mirror an inverted U-shaped curve, a model that has also been tested by several other studies (Williams and Goldman-Rakic, 1995; Gjedde et al., 2010). Thus, optimal signaling may depend on the levels of estrogen to best interact with dopamine levels in the median range for optimal striatal function and optimal performance during such a task.

This hypothesis has been tested in humans. Estrogen-DA interaction in PFC function during a working memory task has been linked to variations in the gene for catechol-o-methyltransferase (COMT), the enzyme that metabolizes synaptic dopamine (Jacobs and D'Esposito, 2011). The authors found *val/val* women to perform poorly with low estrogen levels (early follicular phase) and improve with rising estrogen levels (late follicular phase), whereas *met/met* women show the opposite pattern. Best performers were women with high COMT (low DA) just prior to ovulation (high estrogen levels), and women with low COMT activity (high DA) during menses, further supporting the inverted U-shaped action of DA. Based on these findings, the authors propose that the effect of estrogen on cognitive performance could be either beneficial or detrimental depending on COMT genotype and COMT enzymatic activity (Jacobs and D'Esposito, 2011). While these concepts require further testing, they offer interesting perspectives for the planning of HRT in postmenopausal women.

Several neuropsychiatric pathologies that display a substantial sexual dimorphism have been linked to abnormal dopaminergic function, such as schizophrenia (Brunelin et al., 2013), Parkinson's (Dluzen and Horstink, 2003; Horstink et al., 2003; Sanchez et al., 2010) or Alzheimer's disease (Reeves et al., 2009). Thus, a better understanding of the interaction between sex hormones and dopaminergic neurotransmission could help to improve pharmacological treatment regimens for these diseases and significantly impact women's mental health.

### Sex hormones and serotonin interaction

The serotonergic system serves a multitude of roles, most prominently balancing mood (Martinowich and Lu, 2008). Several serotonergically mediated physiological functions are tightly linked to steroid hormones such as sexual behavior or stress response (Biegon, 1990). The highest expression of serotonergic neurons can be found in the dorsal and medial raphe nuclei of the midbrain with ascending fibers projecting to frontal cortex, striatum, thalamus, amygdala, hypothalamus and hippocampus (Figure 1D). Serotonin (5-HT) receptor subtypes belong to G-protein coupled or ligand-gated ion channels and 15 different subtypes have been characterized (Table S2), which is why disentangling specific neurochemical action for each subtype has been described as complex (Murphy et al., 1998b).

It is also difficult to provide a detailed characterization of serotonergic action in the CNS and how it can be influenced by sex hormones. A multitude of factors plays into serotonergic neurotransmission such as: endogenous levels of serotonin, more than 15 neuroreceptor-subtypes, individual receptor expression, binding and affinity, the functional polymorphism of the serotonin transporter (5-HTT), intracellular protein levels, synthetic enzymes, and the prominent sexual dimorphism (Rubinow et al., 1998). The 5-HTT transports 5-HT back from the synaptic cleft into the presynaptic neuron, where the neurotransmitter is predominantly metabolized by Monoamine Oxidase A (MAO-A). Furthermore, the serotonergic system is a main target of steroid hormones, cytokines, neuropeptides and trophic factors, all of which impact the generation and efficacy of serotonergic neurotransmission (McEwen, 2002; Bethea and Reddy, 2012).

Estrogen has been reported to have potent serotonin-modulating properties from the level of neurotransmitter synthesis via the regulation of tryptophan hydroxylase (Lu et al., 1999) and degradation of 5-HT to the density and binding of 5-HT receptors (Bethea et al., 2002). The effect of estrogen on serotonin expression seems to depend on several factors such as: receptor subtype, brain area and in case of estrogen treatment also on the duration of treatment. On the one hand, estrogen administration has been found to increase tryptophan hydroxylase mRNA (TPH, serotonin synthesizing enzyme) (Pecins-Thompson et al., 1996; Berman et al., 2006); 5-HT_2A_ mRNA levels in brain areas relevant for the control of mood, mental state and cognition (Sumner and Fink, 1998) and 5-HTT mRNA when administered for a longer period (Smith et al., 2004). On the other hand, estrogen treatment has also been observed to decrease mRNA related to serotonergic neurotransmission. For instance, 5-HT1B autoreceptor mRNA in dorsal raphe (Hiroi and Neumaier, 2009) and MAO-A mRNA and activity (Gundlah et al., 2002) are decreased after estrogen treatment. Furthermore, acute estrogen administration decreases 5-HTT mRNA levels (Pecins-Thompson et al., 1998) and 5-HT_1A_ mRNA levels and binding. The latter effect disappears after a more chronic treatment-regimen (Osterlund et al., 1999, 2000). Thus, assigning the effects of estrogen on serotonin to a homogenous functional class of stimulation or inhibition seems not to be feasible.

Progesterone has been suggested to increase serotonergic neurotransmission via the regulation of the expression of serotonin-related genes and proteins (Bethea et al., 2002; Smith et al., 2004; Sanchez et al., 2005). Chronic progesterone treatment seems to decrease 5-HT_1A_ receptor expression in rats (Biegon et al., 1983), a finding that has also been reported for progesterone in combination with estrogen (Henderson and Bethea, 2008). In this study, the authors showed a decrease of 5HT_2C_ receptor expression when progesterone was added to the estrogen administration in macaques (Henderson and Bethea, 2008).

Both, estrogen and progesterone have been demonstrated to modify the serotonergic responsivity to selective serotonin reuptake inhibitors (SSRI)-administration (Benmansour et al., 2012). Experiments in rhesus macaques suggest that the sex steroids interact with the functional polymorphism of the 5-HTT to influence SSRI treatment response (Michopoulos et al., 2011). In humans, a recent study found an association between functional polymorphic region of the serotonin transporter gene (5-HTTLPR) and antidepressant efficacy in non-menopausal women (Gressier et al., 2014). Non-menopausal women with at least one copy of the long allele showed better antidepressant efficacy than those who were homozygous for the short allele (Gressier et al., 2014). Intriguingly, no differences were found in menopausal women. Menopause is typically associated with estrogen withdrawal suggesting that the hormonal status is critical for antidepressant efficacy. These findings add to previous studies suggesting that menopausal women gain less benefit from antidepressant treatments compared to women during their reproductive years (Pinto-Meza et al., 2006; Pae et al., 2009). In conclusion, the interaction between ovarian hormone levels, age and genotype appear to modulate serotonergic reactivity in females.

## Influence of sex steroid hormones on neural circuits: from health to vulnerability and disease

### The impact of sex hormone fluctuation on the healthy brain

Several lines of evidence from neuroimaging indicate modulatory effects of sex steroid hormones on different structural and functional brain connectivity parameters such as white matter structure (De Bondt et al., 2013), gray matter structure (Protopopescu et al., 2008), and overall network connectivity (Hausmann et al., 2002; Weis et al., 2008; Weis and Hausmann, 2010; Thimm et al., 2014).

On the overall brain level, it has been proposed that ovarian hormones facilitate both cortico-cortical and subcortico-cortical functional connectivity, whereas testosterone seems to decrease subcortico-cortical functional connectivity, but increases functional connectivity between subcortical brain areas, as reviewed by Peper et al. (2011). In line with this hypothesis, high levels of endogenous estradiol and progesterone have been observed to raise functional communication between both hemispheres (Hausmann et al., 2002), a mechanism that has been speculated to underlie sex differences in functional cerebral asymmetries (FCA) (Hausmann et al., 2002; Weis and Hausmann, 2010).

Ovarian hormone levels fluctuate on a monthly basis in women. Thus, studying functional and structural brain organization across the menstrual cycle represents a feasible approach to address the question whether sex hormones can influence functional and structural connectivity. A voxel-based morphometry (VBM) MRI study found gray matter density to increase in the right anterior hippocampus and decrease in the right dorsal basal ganglia in the late-follicular phase compared to late luteal phase (Protopopescu et al., 2008). Pletzer et al. report significant differences in gray matter density between naturally cycling women and women using oral contraceptives (OC), observing increased gray matter volume in prefrontal and temporal regions in OC users (Pletzer et al., 2010). Furthermore, the potential impact of hormonal contraception on brain structure does not seem to be limited to gray matter, and white matter tracts seem to be altered by OC use as well, especially in the fornix (De Bondt et al., 2013).

Sexual dimorphism in functional networks of the brain, such as the default mode network (DMN), a network that is proposed to underlie physiological processes unrelated to any particular thought (Gusnard et al., 2001), has been well established (Peper et al., 2011; Tian et al., 2011). However, few neuroimaging studies have investigated the extent to which sex hormones can influence the behavior of functional networks at rest. A recent study exploring functional connectivity of the anterior DMN and the executive control network (ECN) found differences in intrinsic connectivity between OC users and naturally cycling women, and it reported connectivity to differ most between groups in the left angular gyrus, the middle frontal gyrus, and the anterior cingulate cortex (ACC) (Petersen et al., 2013). Within the group of naturally cycling women, the follicular phase was associated with an increase in connectivity with the ECN relative to the luteal phase in the right ACC (Petersen et al., 2013).

A better understanding of functional and structural connectivity changes in the context of sex steroid fluctuations seems crucial to establish neurobiological models of neuropsychiatric diseases that display a strong sexual dimorphism, such as depression (Kessler et al., 1993; Kessler, 2003). Linking functional connectivity measures to neurochemical mechanisms during hormonal transition periods can be viewed as one of the next frontiers in the field of neuroimaging.

### How sex hormone fluctuation can represent a period of heightened risk for the brain: from vulnerability to disease

Women have a lifetime prevalence rate for depression 1.5–3 times higher than men (Kessler, 2003). This distinction between the sexes is most prominent during the reproductive years (Soares and Zitek, 2008). For women, hormonal transitions across their lifespan represent periods of elevated vulnerability to development of mood disorders: elevated and fluctuating sex hormones seem to predispose women to mood-disturbance, beginning with a heightened risk of developing a depressive episode following puberty (Soares and Zitek, 2008).

Pregnancy has been speculated to offer some protection against depression (Ko et al., 2012), including findings of lower suicide rates during pregnancy (Hawton, 2000; Oates, 2003). However, other work suggests that there is no difference in the prevalence rates of depression between pregnant and non-pregnant women (Vesga-Lopez et al., 2008). In a recent extensive study that screened 10,000 postpartum women, one third of screen-positive postpartum depressed women reported the onset of their depressive symptoms during pregnancy (Wisner et al., 2013). Pregnancy has also been discussed to confer vulnerability upon women who are already at risk of developing a depressive illness (Cohen et al., 2004, 2006b). Furthermore, recent findings (Rallis et al., 2014) indicate that symptom levels of depression, anxiety, and stress vary over the course of pregnancy, with women experiencing fewer symptoms during the middle of the pregnancy. Increased depressions scores early in pregnancy seemed to be predictive of later depression symptoms, especially postbirth (Rallis et al., 2014).

In summary, the current evidence does not unequivocally support pregnancy itself to pose an increased risk of developing depression, nor does it clearly identify this period as protection for the majority of women from mood disorders. Factors that might contribute to this ambiguity include that clinical assessment of depression during pregnancy and the postpartum period is complicated, because many of the typical depressive symptoms (disruption of sleep and appetite) are unavoidable during pregnancy and postpartum (Marcus, 2009), and that there may be a distinct pattern of reactivity in women who have never had a depressive episode before, vs. women who are already at risk (Cohen et al., 2004, 2006b).

To better address these challenges in the future, several strategies could be implemented: (1) A routine and serial screening process for depressive symptoms, administered by trained midwives, throughout pregnancy, and during the immediate and the extended postpartum period that focuses on the psychological symptoms, such as changes in mood, the tendency to ruminate, and anxiety. Studies suggest that screening with simple checklists and short screening tools, such as the Edinburgh Depression Scale, can already result in highly efficient screening for postpartum depression (PPD) (MacArthur et al., 2002). (2) Standardized psychoeducation on depression during pregnancy, postpartum blues, and PPD needs to become an integral part of standardized prenatal and postpartum care to help destigmatize PPD and facilitate the process of seeking proper diagnosis and adequate treatment. (3) Based on converging data from 17,000 women, individually tailored psychosocial and psychological intervention is a very promising way to prevent PPD, with provision of intensive, professionally-based postpartum home visits, telephone-based peer support, and interpersonal psychotherapy among the most effective strategies (Dennis and Dowswell, 2013). Thus, within the psychoeducational process, emphasis should be placed on individual risk evaluation, as well as on the wide range of intervention options available to women who fall within the spectrum of pregnancy-related or postpartum mood disorders, covering aspects from psychosocial support, psychotherapy, and psychopharmacology.

In this section, we focus on three examples of hormonal transition across the adult female lifespan: (a) the postpartum period, (b) the perimenopausal period, and (c) the more subtle fluctuation of sex hormones during the menstrual cycle.

#### Postpartum period

With the loss of the placenta, estrogen levels decrease 100–1000-fold during a period of a few days (Nott et al., 1976; O'Hara and Swain, 1996; O'Hara et al., 2000). This dramatic hormonal change is likely to induce a cascade of signaling that also affects the brain. A PET study investigating the neurochemistry of the female brain in the immediate postpartum period found a substantial whole-brain increase in MAO-A in the brain in the first week postpartum compared to women who had not recently been pregnant (Sacher et al., 2010). MAO-A is an enzyme that metabolizes monoamines, such as serotonin, dopamine and noradrenaline. A significant increase in MAO-A has been proposed to be predictive for the recurrence of major depressive disorder (Meyer et al., 2006). These findings in humans are in line with the inverse relationship between estrogen levels and MAO-A which has been observed in cell lines (Ma et al., 1995), as well as in rat (Luine and McEwen, 1977; Chevillard et al., 1981) and macaque (Gundlah et al., 2002; Smith et al., 2004) models. The acute estrogen drop within the first week postpartum has been proposed to trigger the subsequent MAO-A peak that could explain the depressed mood that a majority of mothers experience during this time (Sacher et al., 2010). Elevated MAO-A levels have also been found in prefrontal cortical regions and areas of the ACC in women with PPD and in women who do not meet criteria for a full PPD but report postpartum crying (Sacher et al., 2014). Thus, the interaction between estrogen and MAO-A seems to be a crucial factor in balancing postpartum mood. Given the current lack of prevention strategies for PPD, translation of biological concepts to facilitate the normalization of MAO-A levels in the brain, including potentially attenuating the acute hormonal withdrawal that can precede such an MAO-elevation, represents a promising line of research.

Further evidence that monoaminergic imbalance contributes to the development and/or severity of PPD symptomatology stems from neurochemical investigations of the serotonergic and the dopaminergic system: similar to depressed patients, PPD-patients with a history of non-postpartum depressed episodes seem to display a decrease in 5-HT1A receptors in the anterior cingulate and mesiotemporal cortices (Moses-Kolko et al., 2012). Postpartum status and unipolar depression have also been associated with lower striatal D2/3 receptor binding in postpartum and unipolar depressed women compared to healthy women who were not postpartum (Moses-Kolko et al., 2012). In summary, the postpartum period seems to be characterized by several monoaminergic alteration processes that are highly relevant to the regulation of mood and emotional processing.

GABA has also been implicated in the neurobiology of PPD: In a human pilot study, reduced occipital cortex GABA levels have been reported in parallel with decreased allopregnanolone levels during the postpartum period, irrespective of PPD diagnosis (Epperson et al., 2006). In animal models of postpartum depression, abnormalities of GABA receptors (R delta and gamma 2 subunits) have been observed and discussed as being related to the substantial progesterone decline postpartum (Maguire and Mody, 2008). Further work in animal models mimicking the hormonal environment of the postpartum period revealed a characteristic behavioral phenotype with vulnerability to helplessness, increased anxiety, and aggression that has been associated with differences in expression of several key genes, such as 5-HTT, BDNF, GABA-A receptor type 4 (Suda et al., 2008). Data obtained from elegant animal models for PPD based on estrogen withdrawal across parturition (Galea et al., 2001) and exposure to high cortisol levels (Brummelte et al., 2006) support changes in steroid hormonal environment to spark dramatic effects in spatial memory and hippocampal morphology. This work further strengthens the strong argument that can be made for the heightened plasticity of the postpartum brain that seems to be driven by a closely intertwined action between sex hormones and neurotransmitters.

#### Perimenopausal transition

The perimenopausal transition period marks the end of the reproductive years and is commonly defined as a decline of ovarian function based on reproductive endocrine and menstrual cycle changes (Harlow et al., 2003). Three key markers for the onset of perimenopause are: (1) 7-day or more change in the menstrual cycle length; (2) a change in menstrual flow amount or duration, or (3) amenorrhea lasting at least 3 months (Harlow et al., 2003). Moreover, especially the increase in FSH levels beside elevated LH levels seems to be linked to a decline in ovarian function (Harlow et al., 2003). With the loss of ovarian function and the associated fundamental changes in the hormonal environment, it is not surprising that this phase in a woman's life is accompanied by changes in eating (Hirschberg, 2012), metabolism (Wing et al., 1991; Lovejoy et al., 2008), sleep (Guidozzi, 2013), behavior (Copeland et al., 2006), mood (Cohen et al., 2006a), sexuality (Dennerstein et al., 2003), immune response (Gameiro et al., 2010), and cognitive function (Greendale et al., 2012).

The concept that the perimenopausal phase represents a vulnerability period for developing a depressive illness is supported by evidence for a high rate of new-onset major depressive episodes (MDE) during this time (Cohen et al., 2006a; Freeman et al., 2006). In a longitudinal, prospective cohort study, Cohen and colleagues found that women with no lifetime history of depression who enter the menopausal transition earlier have a significant risk of first onset of depression (Cohen et al., 2006a). Strikingly, women with a previous history of depression, who also reported the use of antidepressants, had nearly 3 times higher risk of an earlier perimenopausal transition compared to non-depressed women (Harlow et al., 2003). Thus, there seems to be an inverse relationship between depression and the inception of perimenopause. Early perimenopause increases the risk of severe mood disturbances, while a lifetime history of depression predisposes women to an early onset of perimenopause. Furthermore, studies by Young et al. and Harlow et al. indicate that depressed women have lower estradiol and higher LH and FSH level than non-depressed controls (Young et al., 2000; Harlow et al., 2003). Whether the decline in estrogen levels or the cyclic fluctuation of estradiol level, which may increase in the menopausal transition (Cramer et al., 2002), contribute to the occurrence of depressed mood remain controversial (Joffe and Cohen, 1998; Halbreich, 2000).

Similar to the postpartum estrogen drop, perimenopausal estrogen decline seems to relate to an up-regulation of MAO-A levels in the brain. Rekkas et al. have recently shown greater MAO-A binding in the prefrontal cortex during the perimenopausal transition phase compared with age-matched women during their reproductive years and during menopause (Rekkas et al., 2014). The authors did not find any association between MAO-A binding and physical criteria of perimenopause, such as menstrual cycle length, vasomotor symptoms or plasma follicle-stimulating hormone levels. They did, however, find a significant correlation between MAO-A binding and the tendency to cry (Rekkas et al., 2014), a psychological symptom that has been found as a subclinical phenomenon to occur during major shifts of sex hormonal environment (Sacher et al., 2010; Dowlati et al., 2014).

Depressive mood during the perimenopausal years may also be of particular relevance for the development of dementia given that depressive episodes have been shown to increase the risk of Alzheimer's disease in later life (Devanand et al., 1996) and that severity of depression has been observed to predict progression from mild cognitive impairment to Alzheimer's disease (Van der Mussele et al., 2014) and cognitive function in patients with central nervous system disease (van Reekum et al., 2000). Estrogen fluctuations during the perimenopausal phase have been discussed to influence mood and cognition via several mechanisms. In addition to promoting anti-oxidative states that can support cell survival, for instance via balancing MAO-A levels (Ou et al., 2006; Fitzgerald et al., 2007), estrogen has been reported to trigger increased 5-HT_2A_ receptor binding that has been speculated to reduce the amount of β-amyloid deposition, a marker for Alzheimer pathology (Nitsch et al., 1994). Thus, it could be hypothesized that the perimenopausal drop in estrogen decreases the beneficial effect of increased serotonin binding on β-amyloid deposition. In ovariectomized rats, acute administration of estradiol seems to have an antidepressant effect via slowing extracellular serotonin clearance involving ERβ and G-protein coupled receptor. Beyond this, estrogen can also block the effect of SSRIs at the 5-HTT via estrogen receptor alpha (Benmansour et al., 2012).

Until today, it has been an area of much debate whether women with depressive symptoms should be treated with HRT, antidepressants or both. Core menopausal symptoms are related to deficits in declarative memory (Woods et al., 2000), fine motor coordination (Bayer and Hausmann, 2010), and feelings of depression (Hay et al., 1994; Freeman et al., 2006) and anxiety (Kessler, 2003; Faravelli et al., 2013; Soares, 2014). Several reviews and meta-analysis suggest small positive effects of HRT on verbal memory, attention, and reasoning (Hogervorst et al., 2000; LeBlanc et al., 2001; Rice and Morse, 2003; Weber et al., 2014). To generate a positive effect on cognition, the age of HRT onset seems to be crucial. Women receiving HRT earlier seem to improve their cognitive performance compared to either older HRT-treated women or untreated women (MacLennan et al., 2006). This report is supported by neuroimaging findings suggesting increases in hippocampal size following estrogen treatment in postmenopausal women (Eberling et al., 2003). Animal studies further emphasize the age-related effects of estrogen treatment: Adams and colleagues report an up-regulation of both NMDA receptors and dendritic spines on CA1 pyramidal neurons in the hippocampus of young adult female rats, while aging rats respond to estrogen with the up-regulation of the NMDA receptor R1 subunit expression, solely (Adams et al., 2001). This neurochemical mechanism might be a starting point to understand the increased vulnerability of the aging hippocampus and decreased efficiency of HRT when administered later in menopausal transition or menopause and subsequently attenuated cognitive performance. Furthermore, perimenopausal estrogen depletion and greater activity of MAO-A are risk factors for Alzheimer's disease (Burke et al., 2004). When estrogen therapy is used early in menopausal transition, it can protect against dementia (Zandi et al., 2002).

To which extent mood is affected by HRT remains controversial. Estrogen has multifaceted neuromodulating effects and a particular emphasis has been placed on the interaction with the serotonergic system for some of the potential antidepressant benefits estrogen use in HRT may have for women during specific windows of time: Some studies suggest that estrogen might be useful to target perimenopausal (Schmidt et al., 2000; Soares et al., 2001), but not postmenopausal (Morrison et al., 2004).

Evidence from neuroimaging findings to link estrogen and the serotonergic system in humans are still relatively sparse. Animal data support ovariectomy to decrease 5-HT_1_ binding (Biegon and McEwen, 1982), 5-HT_2A_ binding and expression (Sumner and Fink, 1993), and 5-HT transporter binding sites and expression (McQueen et al., 1997; Sanchez et al., 2013). These findings have been shown to be reversible with estrogen replacement therapy. However, interpretation of these animal data is constrained by the fact that they demonstrate specificity according to species, a certain brain area and a specific neurodevelopment stage. In humans, a recent PET study found no significant 5-HT_1A_ binding changes in postmenopausal women after estrogen or combined estrogen/progesterone treatment in any of the investigated brain regions including amygdala, ACC, hippocampus and prefrontal cortex (Kranz et al., 2014). A short course of estrogen can increase cortical 5-HT2A receptor density in healthy postmenopausal women's prefrontal regions (Kugaya et al., 2003). However, it still remains unclear to what extent these receptor density changes relate to clinical outcome as this study was done in healthy women and although the increase of 5-HT2A density was paralleled by improved performance in verbal fluency and in the trail making task, no significant changes in mood was observed (Kugaya et al., 2003). In contrast to short time estrogen administration, long-term estrogen treatment seems to be associated with lower 5-HT_2A_ receptor availability in hippocampus (Compton et al., 2008), a finding speculated to reflect increased activity within the serotonergic system leading to a down-regulation in postsynaptic 5-HT_2A_ receptor density. Again, the behavioral interpretation of these findings is difficult as a negative correlation between 5-HT_2A_ receptor availability and memory performance in postmenopausal females using long-term estrogen treatment (ET) has been found while the assessed depression scores did not differ between postmenopausal ET never-users and ET users (Compton et al., 2008). So far, no human *in vivo* PET study has investigated the effects of HRT on 5-HT-transporter binding. More translational work needs to be carried out before a conclusion regarding the role of estrogen replacement therapy as a potential mood enhancing strategy can be reached.

In summary, promising therapeutic approaches to improve perimenopausal mood and to counter depressive symptoms include strategies like inhibiting MAO-A, increasing multiple monoamines with antidepressants, and administering dietary amino acids that are precursors for the monoamines metabolized by MAO-A (Rekkas et al., 2014; Sacher et al., 2014). Regarding cognition, the use of HRT remains controversial. Many studies report only small effects on cognition such as verbal memory and attention (Hogervorst et al., 2000; Weber et al., 2014). These small effects on cognition and the potential side effects of HRT have to be carefully weighted. Unopposed estrogen cannot be used for extended periods due to the increased risk of endometrial hyperplasia (Furness et al., 2009) and malignancy (Weiderpass et al., 1999). To counter these potential side effects, progestin is normally added in oral contraceptive pills and HRT for endometrial protection (Weiderpass et al., 1999). However, the added progestin may worsen mood in some women (Backstrom et al., 2011). A concept also warranting further research is the targeting of estrogen receptors in a tissue-specific way using selective estrogen receptor modulators (Gambacciani, 2013; Mirkin et al., 2014). In conclusion, therapeutic use of HRT should be carefully considered in the context of perimenopausal symptom severity, age and prior history of HRT, dose, treatment combination, and timing of administration.

#### Menstrual cycle associated mood disturbances

The spectrum of severity in mood fluctuations throughout the menstrual cycle is wide and ranges from reports of less well-being in the premenstrual phase to severe clinical data on suicidal behavior: an evaluation of 44 studies in fertile women found a positive correlation between suicide attempts and menstrual phases that are characterized by low estrogen levels (Saunders and Hawton, 2006). In healthy women, some studies report negative premenstrual changes in mood as common and suggest that the majority of women of reproductive age describe a cycle-dependent increase in negative emotions, such as irritability, impulsivity, fear, and low mood (Halbreich et al., 2007), while other authors claim that there is no substantial evidence for any specific premenstrual negative mood syndrome in the general population (Romans et al., 2012). A subgroup of women, however, suffer from clinical levels of premenstrual mood changes called premenstrual dysphoric disorder (PMDD), a condition that has recently been included in the DSM-V (Epperson et al., 2012a). PMDD core symptoms include anxiety, irritability and depressed mood (Epperson et al., 2012a). Symptoms occur on average 2–3 days before onset of menses and resolve after the onset of menstruation (Backstrom et al., 2003). PMDD symptoms are limited to ovulatory menstrual cycles when the corpus luteum is present (Yen et al., 2013), thus it is reasonable to assume that female gonadal hormones play a causative role. However, no consistent differences in hormonal fluctuations during the menstrual cycle between women experiencing clinical level PMDD and normal controls have been found (Backstrom et al., 2003).

Although the levels of sex hormones do not differ between PMDD and healthy women, there might be an altered genetic susceptibility to affective dysregulation induced by normal sex hormone levels. Preliminary genetic findings state an association between allelic variants in the estrogen receptor alpha gene (ESR2) and PMDD (Woods et al., 2000). As PMDD is a heritable disorder with non-Mendelian pattern (Bayer and Hausmann, 2010), elucidating the underlying genetic variations and the multiple interacting genes that confer increased susceptibility may improve our understanding of how PMDD symptoms develop.

Evidence for an interaction between the altered ESR2 in PMDD and catechol-o-methyltransferase (COMT) Val/Val genotype has been reported in a human haplotype analysis of 91 women with PMDD and 56 controls (Woods et al., 2000). COMT is an enzyme involved in multiple functions, such as estrogen metabolism (Hay et al., 1994) and has been hypothesized to tune prefrontal cortical activation through the regulation of dopamine levels (Belelli et al., 2006). The Val/Val genotype has been associated with decreased dopamine levels in the PFC and tuning efficiency (Soares, 2014). Thus, the authors speculate that a Val/Val genotype accompanied by an ESR2 variation might be a factor that could increase the susceptibility toward a dysphoric state via decreased PFC efficiency and disinhibited subcortical activity. Replication of this finding in a larger sample size, as well as the implementation of a neuroimaging protocol to explore the PFC-amygdala circuit in parallel with a detailed assessment of PMDD symptoms will be needed to further test this hypothesis.

While the magnitude of hormonal fluctuation does not seem significantly altered in women suffering from PMDD, an altered brain response to normal hormonal fluctuation could explain the changes in mood and behavior. Several lines of evidence support this concept: preliminary *in vivo* evidence from a small pilot (PET) study in five women with PMDD has shown that relative to healthy controls, women with PMDD experience a smaller change in 5-HT-receptor 1A binding throughout the menstrual cycle (Jovanovic et al., 2006). In animal models of hormonally induced depression via progesterone withdrawal, depression-like behavior can be modulated through specific serotonergic mechanisms or receptor subtypes respectively. Li and colleagues report that activation of 5-HT_1A_ receptors or inhibition of 5-HT_3_ receptors rapidly decreases immobility in the forced swim test (FST), a prominent model for assessing antidepressant-like behavior in rodents. The FST differentiates between active (swimming and climbing) and passive (immobility) behavior when rodents are forced to swim in a cylinder with no escape options. Conversely, blocking 5-HT_1A_ receptors, activating 5-HT_3_ receptors, or 5-HT_7_ receptors increased depression-like behavior in rats in the FST (Li et al., 2013).

The 5-HTT mediates the recapture of serotonin from the synaptic cleft back into the cell. It is the therapeutic target of the currently most widely prescribed class of antidepressants: the SSRI. SSRIs have been found to be more effective in treating premenstrual symptoms than other non-SSRI drugs or a placebo (Dimmock et al., 2000; Shah et al., 2008). It is of particular interest that the pattern of response to drug therapy is different in patients with PMDD compared to patients suffering from a MDE. PMDD patients respond within the first menstrual cycle to SSRI-treatment (Halbreich and Kahn, 2003), suggesting that an imbalance in the serotonergic system may be of particular relevance to the development of PMDD symptoms.

In the context of serotonergic alternations in PMDD, BDNF has also been implicated. Depression has been shown to be associated with decreased BDNF expression, which can be reversed by antidepressant treatment (Lopez et al., 2013). In a study by Oral and colleagues, PMDD women are associated with increased BDNF levels and increased heat-shock protein 70 (HSP70) levels in the luteal phase compared with controls (Oral et al., 2013). These findings seem contrary to the previous findings by Lopez et al. However, Oral et al. discuss the possibility that increased HSP70 levels, as a molecular defense mediator against proteotoxic stress, might reflect cellular distress in PMDD women and that the respectively increased BDNF levels could be a compensatory mechanism potentially leading to resolved PMDD symptoms in the follicular phase. These compensatory mechanisms seem to fail in depressed patients. Findings of a recent study suggest a relationship between a specific BDNF polymorphism (BDNF Val66Met) and impaired fronto-cingulate cortex activation in response to an emotion processing task displaying angry or fearful emotions in the luteal phase of PMDD women (Comasco et al., 2014). As this interaction just appears to be present in the luteal phase, Comasco and colleagues suggest declining progesterone levels to trigger this phenomenon and discuss these changing progesterone levels to act via direct or chloride pump-mediated influence of BDNF on the GABAergic system. Furthermore, the BDNF Met allele lowers the sensitivity to 5-HT signaling (Martinowich and Lu, 2008), which may influence antidepressant efficacy in PMDD women (Comasco et al., 2014). Martinowich and Lu hypothesize that an increase in extracellular 5-HT, for instance after SSRI use, might increase BDNF levels because inhibition of 5-HTT facilitates serotonergic transmission through 5-HT_4,6,7_ receptor subtypes (Martinowich and Lu, 2008).

Progesterone withdrawal associated with allopregnanolone increase in the luteal phase of the menstrual cycle has been hypothesized to be implicated in PMDD (Backstrom et al., 2011, 2014). Allopregnanolone is known for its similarities with benzodiazepines (Majewska et al., 1986), which can cause drowsiness, poor concentration, and memory impairment (Holbrook et al., 2000). Therefore, heightened allopregnanolone levels have been hypothesized to exhibit similar effects in the brain (Backstrom et al., 2014). Contrary to this hypothesis, a study by Girdler and colleagues found lower luteal phase allopregnanolone levels in PMDD patients with higher anxiety and irritability scores (Girdler et al., 2001). Furthermore, greater luteal phase allopregnanolone concentrations have been shown to be associated with improved symptom ratings in PMDD patients (Wang et al., 1996). A possible explanation for these unexpected findings has been proposed by Backstrom and colleagues: PMDD symptom severity seems to be related to allopregnanolone serum concentration in an inverted U-shaped curve (Backstrom et al., 2011). Negative mood symptoms occur when the serum concentration of allopregnanolone is similar to endogenous luteal phase levels, while low and high concentrations have less effect on mood (Backstrom et al., 2014). This recent hypothesis is extended by the suggestion that negative mood symptoms in women with PMDD could be caused by an increased GABA_A_ receptor sensitivity to allopregnanolone (Backstrom et al., 2014). Allopregnanolone levels have also been reported to increase in the brain after acute and chronic treatment with SSRIs (Lovick, 2013), providing evidence for a direct or indirect connection of allopregnanolone with the serotonergic system. The mechanism by which SSRIs increases allopregnanolone levels is thought to involve direct stimulation of 3α-hydroxysteroid dehydrogenase (3α-HSD), an important enzyme in the allopregnanolone biosynthesis (Compagnone and Mellon, 2000). Not only the susceptibility of the GABAergic system toward allopregnanolone seems to be altered in PMDD, GABA levels might also be abnormal. For instance, Epperson and colleagues found a reduction in the cortical GABA levels during the follicular phase in those with PMDD compared with healthy controls (Epperson et al., 2002). In healthy women, cortical GABA levels fluctuate across the menstrual cycle with decreasing levels from the follicular phase to the luteal phase, whereas the opposite occurred in PMDD women (Epperson et al., 2002).

## Conclusions and perspectives

Pharmacological and behavioral approaches have been combined to demonstrate the critical role of sex steroid hormones in mediating effects on synaptic plasticity, memory, mood, and cognition. Several studies have taken advantage of available selective pharmacological tools and knockout mice to elucidate the underlying molecular mechanism of the observed behavioral and electrophysiological effects. These underlying mechanisms are complicated. Many of them involve rapid non-genomic action on presynaptic receptors like D1 receptors, NMDA receptors, and GABA_A_ receptors. Furthermore, sex hormones act on multiple levels, simultaneously, as well as the interacting neurotransmitter systems that are largely interwoven.

Depending on the neurotransmitter system, sex hormone can exhibit facilitative, excitatory or suppressive, inhibitory effects on neurotransmission. For instance, progesterone has been shown to suppress the excitatory glutamate response (Hausmann and Gunturkun, 2000) and facilitates GABAergic neurotransmission through its action at GABA_A_ receptors (van Wingen et al., 2008), while estrogen exhibits facilitating effects on glutamate transmission (Smith and Woolley, 2004) and suppresses GABA inhibitory inputs. The promoting effect of estrogen on glutamatergic neurotransmission, especially at NMDA receptors (Gazzaley et al., 1996; Woolley et al., 1997; Adams et al., 2004), is the inciting factor for synaptic plasticity and subsequently learning and memory (Foy et al., 1999). Furthermore, estrogen is known to promote dopamine release in the striatum, which might be mediated by the inhibitory effect of estrogen on GABA release, as dopamine terminals are influenced by GABAergic inputs. Thus, a decrease in inhibitory tone might facilitate DA release. This interaction between excitation and inhibition modulated by sex hormones is a key factor for understanding how sex hormones impact neuronal activity in the brain. Estrogen may produce its mentioned effects on cognition and mood especially through modulation of serotonergic function (Epperson et al., 2012b). Estrogen can increase serotonin levels and decrease 5-HT reuptake (Koldzic-Zivanovic et al., 2004), which allows 5-HT to remain longer in the synaptic cleft and exhibit prolonged effects on postsynaptic receptors.

**Figure 2:**
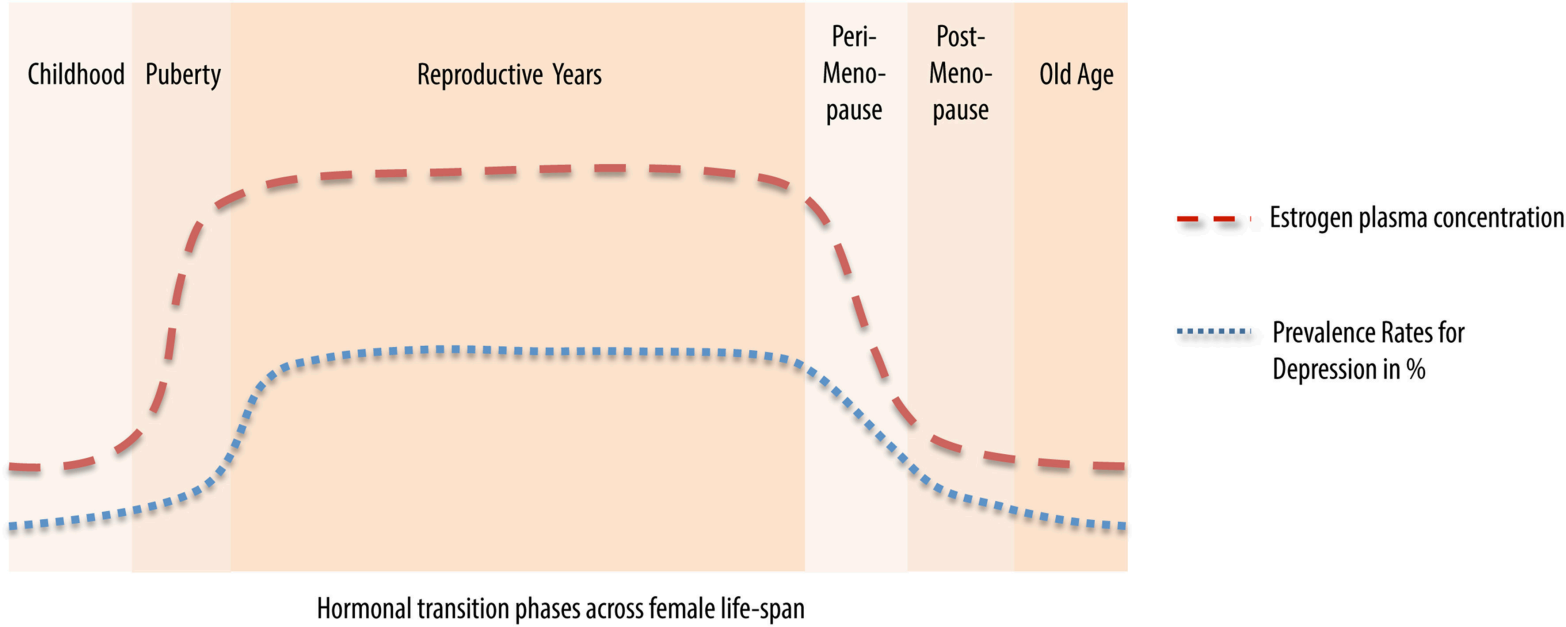
**Vulnerability for development of a depressive illness corresponds to main hormonal transitions across the female lifespan**. During childhood (0–9 years), a phase associated with low estrogen plasma levels, the prevalence rate for depression ranges between 2 and 3% (Kashani et al., 1983; Lewinsohn et al., 1994). When estrogen levels start rising in puberty (10–15 years), so does the prevalence rate for depression, up to 8% (Angold et al., 1998). During reproductive years, a phase when estrogen and progesterone levels peak, prevalence rates vary between 21 and 38% (Kessler et al., 1993; Angold et al., 1998). Estrogen and progesterone levels start declining during perimenopause (41–51 years), drop considerably postmenopausally (45–65 years) and remain fairly stable during old age (above 65 years). This drop in sex steroid levels is paralleled by a decrease in prevalence rates for depression from 23 to 26% (Cohen et al., 2006a; Freeman et al., 2006; Unsal et al., 2011; Tamaria et al., 2013) during the hormonal transition phases to rates of 1–5% during old age (Tamaria et al., 2013).

Variations in hormone levels across the human lifespan exhibit pivotal actions in the human body and seem to heighten the risk of developing certain mood disorder and neurodegenerative pathologies. For women, hormonal transitions such as postpartum, menopause, and subtle fluctuations across the menstrual cycle seem to predispose women to mood disturbance (Figure 2), beginning with a heightened risk of developing a depressive episode following puberty (Soares and Zitek, 2008). Beyond the scope of postpartum or perimenopausal ovarian hormone loss, women can be also be more vulnerable on a monthly basis, across the menstrual cycle. Times of sex hormone withdrawal, as seen before onset of menses, are likely to predispose women to menstrual cycle related diseases such as PMDD.

To summarize, neurotransmitter systems do not work in isolation and sex hormones act on multiple sites, highly intertwined with serotonin, dopamine, GABA and glutamate. Fluctuating hormone levels across the human lifespan may be particularly significant for the etiology of neuropsychiatric diseases that display a prominent sexual dimorphism, such as Alzheimer's disease and depression. A better understanding of the underlying mechanisms that focus on activation of sex steroid specific pathways that could contribute to individual vulnerability to such diseases might allow for more effective treatment and prevention in the future.

Promising approaches include the targeting of estrogen receptors in a tissue-specific way using selective estrogen receptor modulators (Gambacciani, 2013; Mirkin et al., 2014). Further studies are on how treatment responses differ between different hormonal states. Promising future strategies involve the integration of basic with clinical neuroscience research, drawing resources from postmortem work, and innovative animal models for sex hormone associated affective disorders, such as the mouse model for PPD (Galea et al., 2008) to inform human *in vivo* neuroimaging experiments in health and disease. Such human neuroimaging studies could benefit from the application of the recently developed MR-PET hybrid scanners that allows for a combination of fMRI and PET, and could also accommodate real-time pharmacological interventions.

### Conflict of interest statement

The authors declare that the research was conducted in the absence of any commercial or financial relationships that could be construed as a potential conflict of interest.

## Supplementary material

Table S1 **Overview of genomic and non-genomic signaling properties for estrogen and progesterone**. Here, we limit the overview for estrogen-signaling to estradiol (E2), the predominant estrogen during reproductive years for estrogen activity (Weis et al., 2008). This table provides a basic summary of the key features for common genomic and non-genomic estradiol/progesterone signaling, a more detailed review can be found elsewhere (Leonhardt et al., 2003; O'Lone et al., 2004; Marino et al., 2006; Singh et al., 2013).

Table S2 **General classification of main neurotransmitter systems (dopamine, serotonin, GABA, glutamate)**.
