# Supplementary material for: Sex hormones affect neurotransmitters and shape the adult female brain during hormonal transition periods
Source: Front Neurosci. 2015 Feb 20;9:37. doi: 10.3389/fnins.2015.00037 (PMC4335177; doi:10.3389/fnins.2015.00037)
Supplement: Table S1 — Overview of genomic and non-genomic signaling properties for estrogen and progesterone. Here, we limit the overview for estrogen-signaling to estradiol (E2), the predominant estrogen during reproductive years for estrogen activity (Weis et al., 2008). This table provides a basic summary of the key features for common genomic and non-genomic estradiol/progesterone signaling, a more detailed review can be found elsewhere (Leonhardt et al., 2003; O'Lone et al., 2004; Marino et al., 2006; Singh et al., 2013). [file Table1.PDF]

		Genomic (nuclear)								Non-genomic (non-nuclear)	
**Hormones**	**Biosynthesis in the brain (Precursor/ Enzyme)**	**Nuclear Receptor**	**Endo-genous ligand**	**Trans-cription Activation Domain**	**Trans-cription Regulation (direct/in-direct)**	**Co-activators**	**Co-repressors**	**Chaperone**	**Targets**	**Membrane-associated Receptor**	**Signaling pathway**
Estradiol (E2)	androgens/ aromatase[266]	Erα	E2	AF-1 AF-2	ERE/AP1, SP1[9]	SRC/p160, TRAP/DRIP, CBP/ p300[9]	RIP140[267], NCor/ SMRT[268]	Hsp90, Hsp70[269]	PRA[15], oxytocin receptor[270]	mERs (e.g. Erα$_{36,46,66}$[271]/ ERβ)	ERK, cAMP/PKA, PLC/PKAs, Ras/Raf/ MAPK, PI3K/Akt[25]
		ERβ		AF-1 (not functional in humans), AF-2[9]							
Progeste-rone (P)	pregneno-lone/3β-hy-droxysteroid dehydro-genase[272]	PRA	P, DHP, THP[273]	AF-1, AF-2[263]	PRE/ SP1[274]	SRC/p160, CBP/ p300[263]	NCor/ SMRT, HDACs[263]	Hsp90, Hsp70, Hsp40 immunophilin (FKBP51, FKBP52)[269; 275]	PRB, ER, gluco-corticoid receptor[276]	mPRs ($_{α,β,γ,δ,ε}$), PGMRC ($_{1,2}$)[264]	ERK, cAMP/PKA, PKG, Ca^{2+} influx/PKC, PI3K/Akt[25]
		PRB		AF-1, AF-2, AF-3[263]							

Abbreviation: (m)ER = (membrane) estrogen receptor, (m)PR = (membrane) progesterone receptor, DHP = dihydroprogesterone, THP = tetrahydroprogesterone, AF = activation function, ERE = estrogen response element, AP = activator protein, SP = stimulating protein, PRE = progesterone response element, SRC = steroid receptor coactivator, TRAP/DRIP = thyroid hormone receptor associated protein/vitamin D receptor-integrating protein, CBP = CREB-binding protein, RIP = receptor-interacting protein, NCor = nuclear receptor corepressor, SMRT = silencing mediator for retinoid and thyroid receptors, HDACs = histone deacetylases, Hsp = heat-shock protein, PGMRC = progesterone membrane receptor component, ERK = extracellular regulated kinase, PLC = phospholipase C, PKC = protein kinase C, MAPK = mitogen activated protein kinase, PI3K = phosphatidyl inositol 3 kinase
