# Supplementary material for: Sex hormones affect neurotransmitters and shape the adult female brain during hormonal transition periods
Source: Front Neurosci. 2015 Feb 20;9:37. doi: 10.3389/fnins.2015.00037 (PMC4335177; doi:10.3389/fnins.2015.00037)
Supplement: Table S2 — General classification of main neurotransmitter systems (dopamine, serotonin, GABA, glutamate). [file Table2.PDF]

Neuro-transmitter	Biosynthesis (Precursor/Enzyme)	Transporter	Receptor	Major signaling pathway	Regional localization
**Dopamine**	1. phenylalanine/phenylalanine-hydroxylase<br>2. tyrosine/tyrosine hydroxylase<br>3. L-DOPA/aromatic amino acid decarboxylase[277]	DAT[278]	D1-like family ($D_{1,5}$)<br>D2-like family ($D_{2,3,4}$)	↑cAMP<br>↓cAMP	C, STR (CN), SN, AMYG, HC [101; 279; 280]<br>STR (GP), NAcc, OLB[101; 279; 280]
**Serotonin**	1. L-tryptophan/tryptophan hydroxylase<br>2. 5-hydroxy-L-tryptophan/decarboxylase[281]	5-HTT[136]	$5\text{-HT}_{1A\ (D\alpha,\ D\beta,\ E,\ F)}$<br>$5\text{-HT}_{2A\ (B,C)}$<br>$5\text{-HT}_{3}$<br>$5\text{-HT}_{4}$<br>$5\text{-HT}_{5A}$<br>$5\text{-HT}_{6}$<br>$5\text{-HT}_{7}$	↓cAMP<br>IP3<br>ion channel<br>↑cAMP<br>↓cAMP<br>↑cAMP<br>↑cAMP	C, RN, S, AMYG, HC, HTH[282]<br>C, HC, AMYG, NAcc, STR, HTH[283]<br>AMYG, C, HC, NAcc, SN, VTA, BS[284]<br>BG, HC, STR, NAcc, C[285; 286]<br>C, HC, CER, HTH, STR, S[287]<br>STR, HC, C[285]<br>HC, CN, GP, THAL, DRN, SN, SCN, C[285; 288]
**GABA**	glutamate/<br>glutamine acid decarboxylase[64]	GAT_1,<br>GAT_3[289]	$GABA_A$<br>$GABA_B$	ion channel<br>↓cAMP	C, HC, BG, THAL, CER, BS[290]<br>C, HC, BG, THAL, CER, BS[290]
**Glutamate**	glucose-derived<br>tricarboxylic acid cycle intermediates[291]	$EAAT_{1\text{-}5}$[292; 293],<br>$VGLUT_{1\text{-}3}$[294]	NMDA<br>AMPA<br>Kainate<br>Group I $mGlu_{1,5}$<br>Group II $mGlu_{2,3}$<br>Group III $mGlu_{4,6,7,8}$	ion channel<br>ion channel<br>ion channel<br>IP3<br>↓cAMP<br>↓cAMP	Ubiquitous, C, predom. forebrain, HC[295]<br>HC, C[296]<br>HC, C, CER[296]<br>Widespread, HC, C[295; 297; 298]<br>Widespread, HC, C[295; 298]<br>Widespread, HC, C, CER[295; 298]

Abbreviation: + = excitatory; - = inhibitory; GABA = Gamma-aminobutyric acid; L-DOPA = L-3,4-dihydroxyphenylalanine; DAT = dopamine active transporter; 5-HTT = 5-hydroxytryptamine transporter; GAT = Gamma-aminobutyric acid (GABA) transporter; EAAT = excitatory amino-acid transporter, VGLUT = vesicular glutamate transporter; NMDA = N-methyl-D-aspartate; AMPA = α-amino-3-hydroxy-5-methyl-4-isoxazolepropionic acid; mGlu = metabotropic glutamate receptor; IP3 = inositol-1,4,5-trisphosphate; cAMP = cyclic adenosine monophosphate; STR = striatum; CN = caudate nucleus; GP = globus pallidus; NAcc = nucleus accumbens; OLB = olfactory bulb; RN = raphe nucleus (D = dorsal); S = septum; AMYG = amygdala; HC = hippocampus; HTH = hypothalamus; C = cortex; VTA = ventral tegmental area; BS = brain stem; SN = substantia nigra; SCN = suprachiasmatic nucleus; THAL = thalamus; BG = basal ganglia
